# The Impact of Resources for Clinical Surveillance on the Control of a Hypothetical Foot-and-Mouth Disease Epidemic in Denmark

**DOI:** 10.1371/journal.pone.0102480

**Published:** 2014-07-11

**Authors:** Tariq Halasa, Anette Boklund

**Affiliations:** Section of Epidemiology, the National Veterinary Institutes, Technical University of Denmark, Copenhagen, Denmark; INIAV, I.P.- National Institute of Agriculture and Veterinary Research, Portugal

## Abstract

The objectives of this study were to assess whether current surveillance capacity is sufficient to fulfill EU and Danish regulations to control a hypothetical foot-and-mouth disease (FMD) epidemic in Denmark, and whether enlarging the protection and/or surveillance zones could minimize economic losses. The stochastic spatial simulation model DTU-DADS was further developed to simulate clinical surveillance of herds within the protection and surveillance zones and used to model spread of FMD between herds. A queuing system was included in the model, and based on daily surveillance capacity, which was 450 herds per day, it was decided whether herds appointed for surveillance would be surveyed on the current day or added to the queue. The model was run with a basic scenario representing the EU and Danish regulations, which includes a 3 km protection and 10 km surveillance zone around detected herds. In alternative scenarios, the protection zone was enlarged to 5 km, the surveillance zone was enlarged to 15 or 20 km, or a combined enlargement of the protection and surveillance zones was modelled. Sensitivity analysis included changing surveillance capacity to 200, 350 or 600 herds per day, frequency of repeated visits for herds in overlapping surveillance zones from every 14 days to every 7, 21 and 30 days, and the size of the zones combined with a surveillance capacity increased to 600 herds per day. The results showed that the default surveillance capacity is sufficient to survey herds on time. Extra resources for surveillance did not improve the situation, but fewer resources could result in larger epidemics and costs. Enlarging the protection zone was a better strategy than the basic scenario. Despite that enlarging the surveillance zone might result in shorter epidemic duration, and lower number of affected herds, it resulted frequently in larger economic losses.

## Introduction

Foot-and-mouth disease (**FMD**) is a highly contagious viral disease affecting ruminants and pigs [1], [2], [3], and may have a large economic impact on FMD-free countries and regions, in case of an epidemic [4], [5].

Following the FMD epidemic within the European Union (**EU**) in 2001, the European Commission updated a set of regulations and measures to control possible future epidemics of FMD in its member states [6], [7]. The measures include, among others, depopulation of detected herds and establishing 3 km protection and 10 km surveillance zones around them, in which movement restrictions and surveillance of herds are performed. As these measures, however, may not be sufficient to control an expanding or already widespread epidemic, additional control measures must be considered, such as emergency vaccination [7] and/or pre-emptive depopulation [8].

For the national veterinary authorities, the application of protective emergency vaccination insures a public support compared to the mass killing of healthy animals, in case suppressive emergency vaccination or pre-emptive depopulation is applied [6], [9]. Nonetheless, from economic standpoint, protective emergency vaccination seems not to be a recommended control strategy in case of an epidemic in Denmark [10]. Thus the question remains to whether it is possible to minimize the economic loss due to an FMD epidemic in a large exporting country of livestock and livestock products such as Denmark, without the need to kill a large number of animals.

Clinical surveillance of herds within the protection and surveillance zones has the purpose to detect infected herds early, and thus limit the spread of the disease. The effect of enlargement of the zones must depend on whether the spread of disease is limited within the existing zones, or whether the disease is often spread to the area surrounding the zones. It has been shown that the disease can spread from one herd to another over distances longer than 10 km, which is the radius of the standard surveillance zone [11], [12], [13]. This means that enlargement of zones might limit the spread of the disease.

In order to model enlargements of zones and clinical surveillance properly, it is necessary to take into account the available resources for clinical surveillance. Resources can be a limitation, which is necessary to consider in a country that is densely populated with livestock herds, such as Denmark, where the daily number of herds to be surveyed might be larger than the surveillance capacity.

It is therefore important to model clinical surveillance properly, which will allow an assessment of whether the current surveillance capacity is sufficient to survey herds on time as required by the EU [7], and the Danish regulations [14], and to prevent delays that could result in extra economic losses The Danish regulations require all herds, within the surveillance zones, to be surveyed within the first 7 days following the establishment of the zone [14]. Modelling these processes will allow an assessment of whether enlargements of the protection and/or surveillance zones, could limit disease spread and the economic losses.

Simulation models are valuable tools that are used to assist the veterinary authorities in contingency planning [4], [8], [10], [11], [15], [16], [17], [18]. They have also been used to study the potential spread of FMD and to evaluate the effectiveness of potential control strategies during an FMD outbreak [19]. To our knowledge, FMD models have not been used to assist whether surveillance capacity in a country is sufficient to survey herds without delays or whether extra resources are needed. Furthermore, the epidemiological and economic effects of enlarging the protection and/or surveillance zones and the impact of surveillance frequency of herds, in overlapping zones, on epidemic consequences have, to our knowledge, not been investigated before.

The objectives of this research were to assess: 1) whether the current surveillance capacity is sufficient to fulfill the EU and Danish regulations to control a hypothetical FMD epidemic in Denmark, 2) whether enlarging the surveillance and/or the protection zones could minimize the economic losses, using either default surveillance capacity, or extra resources for surveillance, and 3) to determine the impact of surveillance frequency of herds in overlapping surveillance zones on epidemic consequences.

## Materials and Methods

### Study area and population

The study consisted of all Danish cattle, swine, sheep and goat herds in the period from 1^st^ October 2006 until 30^th^ September 2007. This period was chosen to avoid possible influence from the outbreak of bluetongue in Denmark in October 2007. The data included 23,550 cattle herds, 11,473 swine herds and 15,830 sheep and goat herds. For each herd, the herd data included the Danish Herd ID System, referred to as CHR (central husbandry register) number, herd type, UTM geo-coordinates, number of animals, and rate of animal movements from the herd per day. Herds were categorized into 3 categories: cattle, swine, and sheep and goats. Cattle herds were categorized as dairy or non-dairy herds. Swine herds were categorized into 19 different types based on their production type and Specific Pathogen Free (SPF) status [20]. Sheep and goats were grouped and treated equally (referred to as sheep herds throughout the paper), because Denmark has a very limited number of goat herds, and because of the disease dynamics in goat herds are expected to be similar to sheep herds. When a farm included several animal species, each species was given a different ID and set as a different herd on the same location and with the same CHR number. Information about markets was also available, including the UTM geo-coordinates.

The input parameters of the model were based on Danish data, the literature and personal communication from experts, and are available in the supplementary materials of a recent publication [10].

### The simulation model

The model simulated hypothetical spread of FMD between herds in Denmark using the dynamic spatial simulation model DTU-DADS (version 0.140), that runs in the statistical software R (Version 3.0.2) [21], based on daily discrete time events. This is an updated version of the DTU-DADS model (version 0.100) [6], [10], [11], which incorporates changes necessary to model resources for surveillance.

The first change included modelling resources for surveillance of herds within the protection and surveillance zones and for traced herds. A queuing system was added to the model, and herds in the protection zone would be set to queue for surveillance two times, once directly following inclusion in the protection zone, and a second time 21 days later, while herds in the surveillance zones would be set to queue for surveillance one time only, directly after inclusion in the zone. For each day modelled, the daily resources for surveillance would determine the number of herds in the queue that would be surveyed. The rest of the queued herds would wait until resources are available. It was assumed that herds that are within multiple surveillance zones will be visited every 14 days, as long as they are in multiple surveillance zones. When a herd enters a new surveillance zone, while it was not anymore in any zone, 8 days must elapse before the herd would get a new surveillance visit. When a herd was in the queue for >7 days but ≤14 days, the second visit, for herds within the protection zone, was changed from 21 to 14 days after the first visit, while when a herd was in the queue for >14 days, the second visit, for herds in the protection zone, was changed to 7 days later. This was carried out to insure that herds are surveyed before lifting the zones [7], to keep the restrictions on movements from and to the herd, and to bind zones' duration to 30 days, in order to limit potential economic damage due to longer zones duration. Nonetheless, to insure that all herds are visited before lifting a zone, when any herd was in the queue for >21 days, the duration of all zones was extended by the longest time a herd was in the queue. For instance, as soon as a herd was in a queue for 22 days, the zone duration was extended from 30 to 52 days. In case a herd was set in the queue for a second visit, while it was already queuing from a previous visit, only the first visit would be executed.

A group of veterinarians and experts from the Danish Veterinary Authorities came together in 2013, in order to assess the available resources in case of an outbreak in Denmark (Personal communication, Maren Holm Johansen from the Danish Veterinary Authorities). Based on the available resources, it was estimated that it would be possible to survey, approximately, 450 herds per day. This number was used as the default surveillance resources capacity and was changed as explained bellow in the sensitivity analysis.

Detection of infected herds is carried out using 3 processes, which are detection of first infected herd, detection of herds by the farmer (basic detection) and finally detection through surveillance visits (as explained above). In the previous version of the model, detection of the first outbreak was always fixed to day 21 following the infection start [6], [10], [11]. Despite that this was based on actual detection data from the UK and the Dutch epidemics in 2001, variation is expected, and hence the detection of the first outbreak was set using a PERT (Program Evaluation and Review Technique) distribution with 18, 21, and 23 days as a minimum, most likely and maximum values, respectively, based on the sensitivity analysis from a previous study [6].

In the previous version of the DTU-DADS model, all infected herds would be eventually detected using the basic detection. This is not realistic as signs could pass undetected in small herds. Basic detection was therefore modelled based on our previous work using InterSpread Plus (version 2.001.11) [10]. Infected herds would be subjected to a probability of selection of 80%. The selected herds would then be subjected to a Bernoulli process of detection based on probabilities of detection that are based on the number of days following the appearance of clinical signs. These probabilities reflected the basic surveillance (farmers' awareness). Detection following surveillance in the zones was also dependent on the number of days following the appearance of clinical signs within the herd, for cattle and swine herds. Sheep herds were sampled for serological analysis as well, and hence probability of detection, in sheep herds, depended on number of days following infection [10]. Herds that were not detected would be recovered from the disease. Recovery was based on a mechanistic module of within-herd spread built in the DTU-DADS model [11], [15]. When all animals in an un-detected herd were recovered, the herd was considered a recovered herd (infection was not detected).

### Disease spread

The simulation starts with one index herd, which is the first infected herd in the epidemic. Other studies have shown that the index herd does influence the size and duration of the epidemic [11], [16], [20]. To include the variation caused by different index herds, we randomly selected index herds of different herd type and when relevant from areas with different animal densities. The index herds were 1,000 cattle herds located in areas with high cattle density, 1,000 in areas with low cattle density, 1,000 swine herds located in areas with high swine density and 1,000 in areas with low swine density, and 1,000 sheep herds. In total 5,000 iterations were run per scenario.

Spread of infection between herds was simulated through 7 spread mechanisms: 1) direct animal movement between herds; 2) abattoir trucks; 3) milk tankers; 4) veterinarians, artificial inseminators, and/or a milk controllers (medium risk contact); 5) visitors, feedstuff and/or rendering trucks (low risk contact); 6) markets; and 7) local spread.

Based on actual movement data, a rate of movements per day was calculated for each herd. The individual daily movement rate was used as lambda in a Poisson distribution to represent the number of movements per day. Similarly, a rate of abattoir deliveries per day was calculated based on herds' actual data and used in a Poisson distribution to simulate the number of movements to the abattoir per day from the infectious herd. Thereafter, the number of herds visited by an abattoir truck on the way to the abattoir following visit to an infected herd was estimated from a Poisson distribution with a lambda depending on the herd type. Based on milk tank deliveries a lambda was calculated and used in a Poisson distribution, to represent the number of times milk is picked up in dairy herds [10]. Likewise, medium and low risk contacts were simulated, but with different lambdas and risks of infection as presented previously [10].

Because markets in Denmark are restricted to cattle only, an infection spreading from a market can initially affect only cattle herds. The spread via markets would be due to direct movements of infected animal to susceptible herds, or via people and vehicles that had been in contact with the infected animals, and then contacted susceptible herds.

Local spread was defined as infection of susceptible herds within a 3 km radius around the infected herd [10], [17] due to unexplained reasons dependent or independent of human activities, such as rodents, birds and flies, machineries and equipment moved between neighboring herds, and to a limited degree airborne spread. Herds located on the same farm had a daily chance of infection of 95%, when one herd was infected.

When a herd was infected, the disease would spread until the herd was detected, and hence was depopulated. The period from when a herd starts showing clinical signs until it was detected, with basic detection, was dependent on the herd type, e.g. cattle herds were detected faster than sheep herds, because some sheep do not show clinical signs.

### Basic control strategy

After detection of the first infected herd, a set of control measures were applied, representing the basic scenario. These included: 1) depopulation, cleaning and disinfection of detected herds; 2) a 3 days national stand still on direct animal movements in the country; 3) creation of a 3 km protection zone and a 10 km surveillance zone around the detected herds; in which movements between herds and out of the zone were restricted and herds were surveyed one (surveillance zone) or two (protection zone) times before lifting the zone; 4) backward and forward tracing of contacts from and to detected herds. When a herd had received animals from a detected herd, the receiving herd was also depopulated and disinfected, while in case of other kinds of contacts, the herd was surveyed. When a herd was subject to surveillance, the animals were inspected for clinical signs of FMD. Sheep herds were also sampled for serological analysis [10].

The daily animal depopulation capacity was set at 2,400 ruminants and 4,800 pigs [10]. Detected herds had higher priority for depopulation than traced herds. In case of several herds on the same farm, all herds on the farm were depopulated, when one herd was detected.

### Alternative scenarios

The alternative scenarios included enlargement of the protection and/or surveillance zones, with a surveillance capacity of 450 herds a day. The protection zone was enlarged to 5 km, while the surveillance zone was enlarged to 15 or 20 km in different scenarios. Furthermore, scenarios were run combining enlargement of the protection zone to 5 km and the surveillance zone to 15 or 20 km, simultaneously.

### Sensitivity analysis

Surveillance capacity was changed from 450 to 200, 350 or 600 herds per day, to study the impact of surveillance capacity on epidemic course and consequences. Furthermore, to study the impact of enlarging surveillance and protections zones with higher resources for surveillance, the zones were enlarged as explained in the previous section, and the surveillance capacities were increased from 450 to 600 herds per day.

Herds that are located in multiple surveillance zones would be surveyed every 14 days as long as they are in multiple surveillance zones, as explained earlier. A sensitivity analysis was conducted, in which herds were surveyed every 7, 21 or 30 days instead. Sensitivity analysis on other important parameters, such as detection time and risk of infection through the different mechanisms of disease spread, is presented in an earlier publication [10].

### Costs calculation

The costs and losses of the epidemics were calculated as presented previously [10]. Briefly, the direct costs consisted of surveillance, depopulation, cleaning and disinfection, empty stable, compensation, and national standstill costs. The indirect costs included losses incurred from restrictions on exports to EU and non-EU countries (export loss). Total costs were calculated per iteration and their summaries were thereafter calculated.

### Statistical analysis

The alternative scenarios were compared to the basic scenario using epidemiological and economic results. The epidemiological results were duration of epidemics, the numbers of infected herds, number of surveillance visits and the numbers of herds detected from surveillance visits, while economic results included the direct costs, export loss and the total costs.

To test the statistical differences between the scenarios, we used the Wilcoxon rank sum test run in the statistical software R (Version 3.0.2) [21].

## Results

### Basic scenario

Out of the 5,000 iterations that represented the 5 different index herd types, there were 13 iterations in which the epidemics fade out before the disease was detected. All of these epidemics started in small sheep herds. In 11 out of the 5,000 iterations, the duration of the protection and surveillance zones were prolonged to more than 30 days, due to the lack of resources to survey herds within the time limit. Nine of these epidemics started in cattle herds. A large number of the herds that were in surveillance zones were actually in overlapping surveillance zones. For example, there were 1,701 (286–4,692, 5^th^ and 95^th^ percentiles (5–95%)) herds included in 2 or more surveillance zones in epidemics initiated in cattle herds in high cattle density areas, which is 55% (27–79%) of the total number of herds in the surveillance zones.

Epidemics initiated in cattle herds were larger, longer in duration and costlier than epidemics initiated in swine and sheep herds (Table 1). For example, when epidemics were initiated in cattle herds in high cattle density areas, the median epidemic duration was 45 days (14–113 days, 5–95%), the median number of infected herds was 56 (10–192, 5–95%), and the median total costs was €522 million, (€400–€829 million, 5–95%) (Table 1). In total, a median of 11,122 surveillance visits (1,896–35,839, 5–95%) were conducted in herds within the protection and surveillance zones and in traced contact herds.

**Table 1:** Median (5^th^ and 95^th^ percentiles) of epidemic duration, number of infected herds, number of surveillance visits, direct costs, export loss and the total costs of the epidemic, that were initiated in cattle herds in high (**highCat**) and low (**lowCat**) cattle density area, swine herds in high (**highPig**) and low (**lowPig**) swine density area and in sheep herds (**sheep**).

	Epidemic duration (days)^1^	Infected herds	Surveillance visits	Direct Costs (€×10^6^)	Export loss (€×10^6^)	Total costs (€×10^6^)
**highCat**						
** Basic**	45 (14–113)	56 (10–192)	11,122 (1,896–35,839)	31 (10–103)	491 (388–720)	522 (400–829)
** PZ5**	44 (13–110)	56 (9–182)	12,345*^2^ (1,869–35,485)	31 (10–97)	487 (386–718)	519 (398–800)
** SZ15**	43 (13–95)	51* (9–167)	16,125*** (3,089–37,513)	39*** (12–128)	504 (386–743)	544** (399–860)
** SZ20**	41*** (13–92)	48*** (9–165)	17,225*** (3,304–38,697)	44*** (13–193)	506*** (395–842)	551*** (408–1,036)
** PZ5+SZ15**	43 (14–99)	53 (10–175)	16,606*** (3,042–39,932)	39*** (12–139)	502* (388–748)	541*** (402–887)
** PZ5+SZ20**	41*** (13–94)	47*** (9–151)	17,923*** (4,242–40,728)	45*** (14–193)	507*** (388–849)	553*** (404–1,053)
**lowCat**						
** Basic**	57 (17–129)	77 (13–269)	12,746 (1,582–37,561)	34 (10–105)	522 (393–766)	558 (405–858)
** PZ5**	58 (18–131)	77 (13–243)	13,644* (1,928–38,532)	33 (10–101)	524 (394–748)	558 (405–839)
** SZ15**	50*** (16–119)	70** (12–230)	16,817*** (2,217–42,861)	39*** (11–140)	517 (392–793)	556 (405–924)
** SZ20**	50*** (15–113)	65*** (12–223)	19,609*** (2,675–45,737)	45*** (12–193)	525 (396–845)	571*** (409–1,032)
** PZ5+SZ15**	51*** (17–116)	66** (12–238)	17,412*** (2,573–44,438)	38** (11–143)	515 (400–800)	553 (412–933)
** PZ5+SZ20**	50*** (16–117)	64*** (12–235)	19,307*** (2,879–48,430)	44*** (13–203)	521 (399–893)	564*** (415–1,101)
**highPig**						
** Basic**	33 (7–101)	27 (4–129)	4,852 (656–26,873)	18 (8–72)	451 (364–657)	468 (372–726)
** PZ5**	35 (7–98)	28 (4–124)	5,437* (837–26,347)	19 (8–67)	452 (360–659)	469 (369–717)
** SZ15**	32 (7–90)	26 (4–114)	8,074*** (1,283–30,486)	24** (10–83)	453 (366–661)	477* (379–745)
** SZ20**	33 (6–84)	25 (4–102)	10,867*** (1,529–33,674)	28*** (11–104)	466*** (366–700)	494*** (378–802)
** PZ5+SZ15**	33 (7–83)	26 (4–112)	8,513*** (1,281–31,010)	24*** (10–82)	455 (368–644)	476*** (380–722)
** PZ5+SZ20**	32* (7–85)	26 (4–100)	10,456*** (1,746–34,406)	28*** (11–105)	458** (372–699)	486*** (385–803)
**lowPig**						
** Basic**	38 (7–113)	32 (4–158)	5,670 (588–25,611)	18 (7–66)	459 (364–679)	477 (372–743)
** PZ5**	39 (7–108)	31 (4–151)	5,996 (737–27,280)	17 (7–65)	460 (359–673)	479 (367–732)
** SZ15**	33*** (7–95)	28*** (4–114)	7,811*** (1,034–29,805)	21*** (8–73)	451 (361–656)	472 (371–727)
** SZ20**	33*** (8–94)	27*** (4–114)	10,452*** (1,631–34,370)	25*** (8–93)	461 (366–693)	486 (376–770)
** PZ5+SZ15**	33** (8–94)	29*** (4–121)	8,642*** (1,180–30,925)	21*** (8–76)	458 (362–639)	479 (371–718)
** PZ5+SZ20**	31*** (8–92)	25*** (4–117)	10,268*** (1,757–36,752)	24*** (9–100)	455 (371–704)	479 (380–813)
**Sheep**						
** Basic**	30 (2–100)	20 (2–138)	3,341 (365–25,220)	13 (6–70)	435 (346–658)	449 (354–722)
** PZ5**	31 (2–97)	21 (2–126)	3,823* (410–26,260)	14 (6–66)	438 (345–641)	450 (352–710)
** SZ15**	30 (2–87)	20 (2–121)	5,692*** (571–31,770)	17*** (7–81)	440 (352–657)	458* (360–729)
** SZ20**	27 (3–83)	18 (2–114)	7,811*** (795–32,775)	20*** (8–112)	441* (350–710)	463** (360–823)
** PZ5+SZ15**	28 (2–96)	17 (2–124)	5,365*** (632–33,031)	16*** (7–88)	435 (349–683)	452 (357–761)
** PZ5+SZ20**	27* (3–83)	19 (2–108)	7,544*** (821–31,203)	19*** (8–98)	439 (354–680)	460** (363–782)

Basic control strategy as described by Danish and European legislation was modelled (**Basic**), and compared to alternative scenarios, with enlargements of the protection zone from 3 km to 5 km (**PZ5**) and surveillance zone from 10 km to 15 km (**SZ15**) or 20 km (**SZ20**), and a combination of these enlargements.

1 Epidemic duration is calculated from detection of the first herd in the epidemic to the last herd is depopulated.

2 Statistical significance level in comparison to the corresponding variable in the corresponding basic scenario (absence of a star represents a P-value ≥0.05, * represents a P-value <0.05, ** represents a P-value <0.01, and *** represents a P-value <0.001).

**Figure 1:**
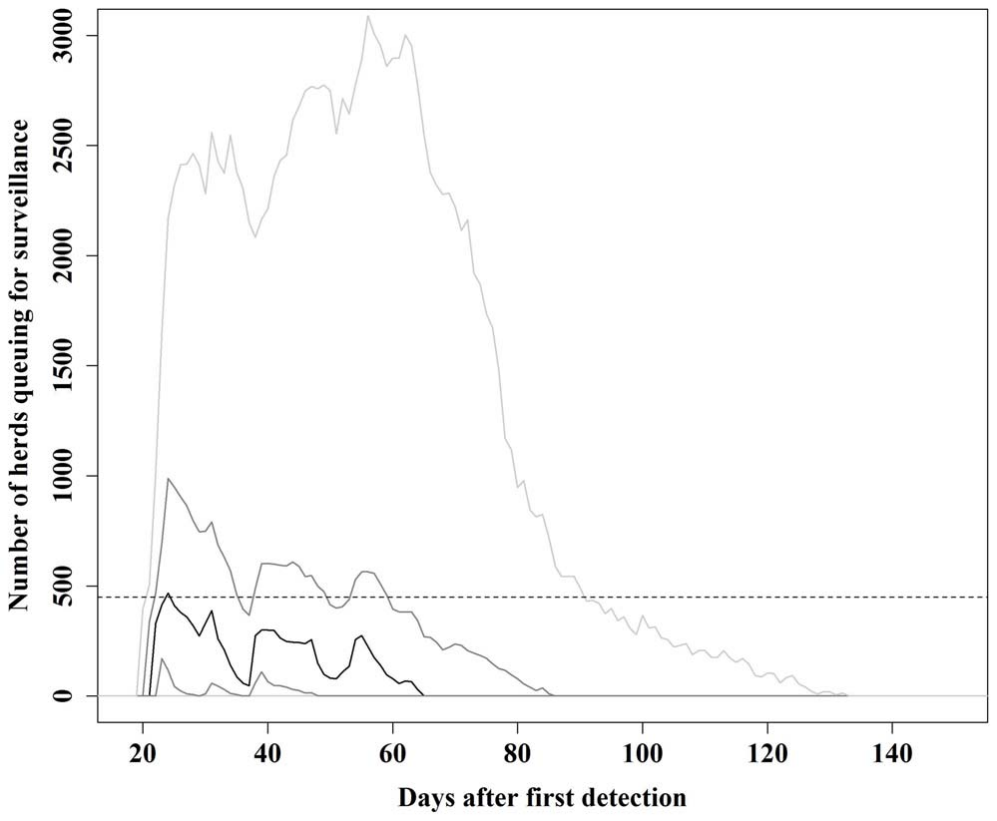
Total number of herds queuing for surveillance visits, for each day, when epidemics were initiated in cattle herds located in areas with high cattle density. A basic control strategy as described by Danish and European legislation was modelled. The black line represents the 50^th^ percentile, the dark gray lines represent the 25^th^ and 75^th^ percentiles and the light gray lines represent the 5^th^ and 95^th^ percentiles. The interrupted line represents the daily surveillance capacity of 450 herds.

**Figure 2:**
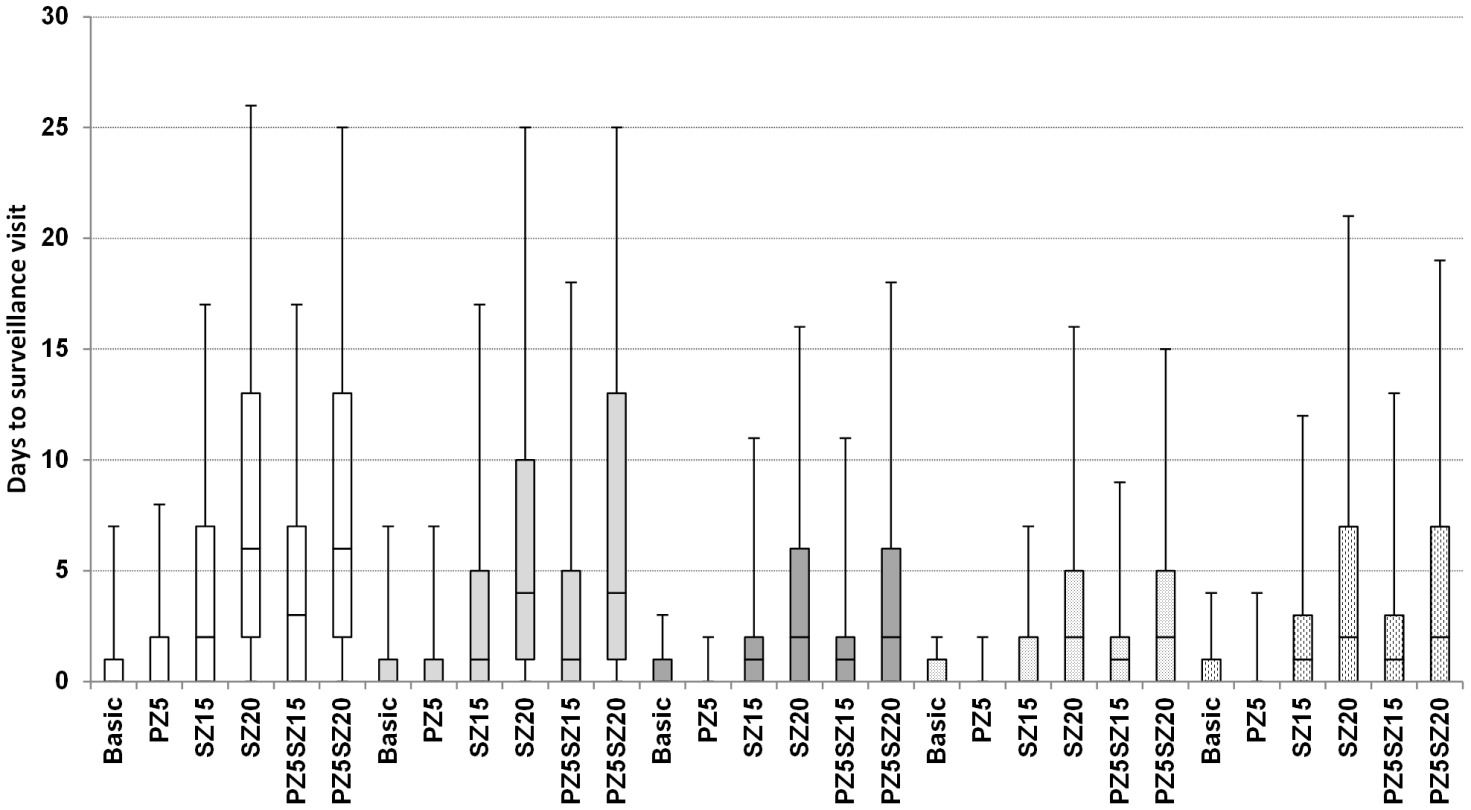
Box plots of the waiting time before a scheduled surveillance visit is executed (days, between a herd was set for surveillance, and until the herd was actually surveyed), in epidemics that were initiated in cattle herds located in areas with high cattle density (empty boxes), cattle herds located in areas with low cattle density (light gray boxes), swine herds located in areas with high swine densities (dark gray boxes), swine herds located in areas with low swine densities (dotted boxes), and in sheep herds (vertical-dashed boxes). A basic control strategy (Basic) as described by Danish and European legislation is compared to alternative scenarios, with enlargement of the protection zone from 3 km to 5 km (PZ5) and of the surveillance zone from 10 km to 15 km (SZ15) or 20 km (SZ20), and a combination of these enlargements. The middle line represents the median, the box represents the 25^th^ and 75^th^ percentiles and the whiskers represent the 5^th^ and 95^th^ percentiles.

For each day, the number of herds queuing for surveillance, in epidemics initiated in cattle herds in high cattle density areas, is shown in Figure 1. It shows that in a median size epidemic, the maximum number of herds queuing for surveillance is approximately 470 herds per day. This means that the available resources (450 herds per day) are sufficient, so that most often herds will be surveyed at the same day they were scheduled for surveillance. In epidemics corresponding to the 75^th^ percentile, the maximum number of herds queuing for surveillance is approximately 990 herds, while in the 95^th^ percentile situation, the maximum number is, approximately, 3,100 herds. In such extreme epidemics, the resources would still be sufficient for surveying herds on time (within 7 days from assignment to surveillance visit) (Figures 1 and 2). Figure 2, shows box plots of the delay time (days between when the herd was set for surveillance and when the herd was actually surveyed) before a scheduled surveillance visit is executed for the basic and alternative scenarios and the 5 different types of the index herds. For the basic scenario, generally, herds would be surveyed at the same day they were set for surveillance. Nevertheless, long delays can occur when epidemics are large, but herds would still be visited on time (Figure 2).

### Alternative scenarios

When the protection zone was enlarged from 3 to 5 km, in 10 of the 5,000 iterations that represented the 5 different types of index herd, the duration of the zones was increased to more than 30 days. When the surveillance zone was enlarged from 10 km to 15 km or 20 km, the number of iterations in which the zone duration was longer than 30 days were 97 and 315, respectively. When the protection zone was enlarged to 5 km and the surveillance zone was simultaneously enlarged to 15 or 20 km, the number of iterations, in which the zone duration was longer than 30 days were 98 and 328, respectively. Prolongation of the zone duration occurred, mainly, when epidemics where initiated in cattle herds.

Enlarging the protection zone from 3 km to 5 km did not change the epidemic duration, number of affected herds and the total costs, regardless the type of index herd that was used to initiate the epidemics (Table 1). However, enlarging the protection zone resulted in the lowest total costs for the 5% worse epidemics (Table 1). Depending on the type of index herd, enlarging the surveillance zone from 10 to 15 km may reduce epidemic duration and the number of infected herds, compared to the corresponding basic scenario, but it would not reduce the economic damage (Table 1). Enlarging the surveillance zone from 10 to 20 km resulted frequently in shorter epidemic duration and fewer infected herds compared to the corresponding basic scenario, especially when epidemics were initiated in cattle herds (Table 1). However, in these situations, larger number of surveillance visits and higher costs were predicted (Table 1). Enlarging the protection zone to 5 km and the surveillance zone to 20 km resulted in the shortest epidemic duration and the lowest number of infected herds, regardless the type of index herd that was used to initiate the epidemics (Table 1). However, this scenario resulted in the largest number of surveillance visits and costs of the epidemics. When the surveillance zone is enlarged, longer delays occurred (Figure 2), due to the larger number of herds queuing for surveillance. This shows that the surveillance capacity would not be sufficient to survey herds on time for large epidemics, and hence extra resources would be needed.

Export losses are the driving force of the total economic losses in general (Table 1), but it also can be seen that the direct costs may increase, when the zones are enlarged, compared to the corresponding basic scenario (Table 1).

### Sensitivity analysis

**Figure 3:**
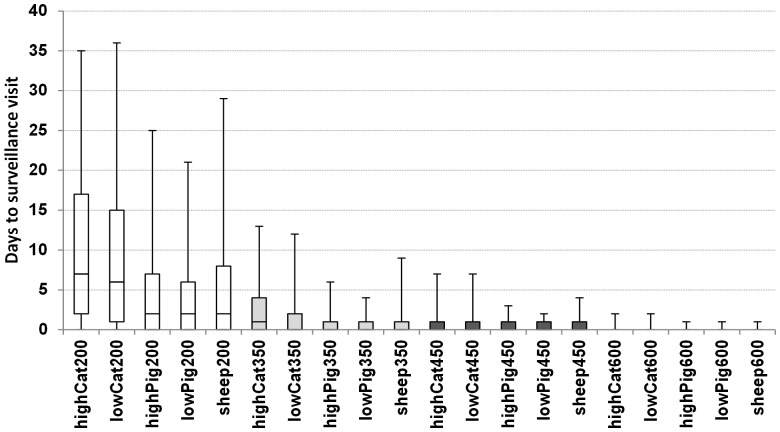
Box plots of the waiting time before a scheduled surveillance visit is executed (difference, in days, between the day the herd was set for surveillance, and the day the herd was actually surveyed), in epidemics that were initiated in cattle herds in high cattle density areas (highCat), cattle herds in low cattle density areas (lowCat), swine herds in high swine density areas (highPig), swine herds in low swine density areas (lowPig), and in sheep herds (sheep). The basic control strategy as described by Danish and European legislation (dark gray boxes) with a surveillance capacity of 450 herds per day is compared to scenarios with reduced or increased surveillance capacity to 200 (empty boxes), 350 (light gray boxes) or 600 (boxes do not appear) herds per day. The middle line represents the median, the box represents the 25^th^ and 75^th^ percentiles and the whiskers represent the 5^th^ and 95^th^ percentiles.

Reducing surveillance capacity would result in longer delay time before a herd is surveyed, while increasing it would result in a shorter delay time (Figure 3). Reducing the capacity to 200 herds per day would result in fewer surveillance visits than the basic scenario (Table 2). However, it might result in longer epidemic duration, larger number of infected herds, and larger economic damage, compared to the corresponding basic scenario (Table 2). Furthermore, it would result in a notably larger variation in the number of infected herds and in the total costs, compared to the corresponding basic scenario (Table 2). Reducing the capacity to 350 herds or increasing it to 600 herds per day did not result in extra economic losses compared to the corresponding basic scenario (Table 2).

**Table 2:** Median with (5^th^ and 95^th^ percentiles) of epidemic duration, number of infected herds, number of surveillance visits and the total costs of the epidemic, using the basic scenario (**Basic**) that represent the EU and Danish control measures, when epidemics were initiated in cattle herds in high (**highCat**) and low (**lowCat**) cattle density area, swine herds in high (**highPig**) and low swine (**lowPig**) density area and in sheep herds (**sheep**); The influence of changes in the surveillance capacity (**Capacity**) from 450 herds per day to 200, 350 or 600 herds per day are compared.

	Epidemic duration (days)^1^	Infected herds	Surveillance visits	Total costs (€×10^6^)
**highCat**				
** Basic**	45 (14–113)	56 (10–192)	11,122 (1,896–35,839)	522 (400–829)
** Capacity-200 herds/day**	47 (14–114)	57 (10–201)	8,822***^2^ (1,785–22,250)	523*** (393–1,030)
** Capacity-350 herds/day**	45 (14–119)	58 (9–197)	11,449 (1,764–32,125)	527 (395–856)
** Capacity-600 herds/day**	46 (14–113)	57 (10–183)	11,171 (1,900–36,460)	528 (400–809)
**lowCat**				
** Basic**	57 (17–129)	77 (13–269)	12,746 (1,582–37,561)	558 (405–858)
** Capacity-200 herds/day**	60 (17–144)	79* (13–297)	10,341*** (1,539–25,940)	564*** (404–1,080)
** Capacity-350 herds/day**	56 (17–133)	76 (14–249)	12,361*** (1,666–32,663)	556 (405–873)
** Capacity-600 herds/day**	57 (17–129)	76 (13–244)	12,514 (1,582–39,502)	559 (402–832)
**highPig**				
** Basic**	33 (7–101)	27 (4–129)	4,852 (656–26,873)	468 (372–726)
** Capacity-200 herds/day**	36* (7–105)	28* (4–146)	4,822*** (678–19,267)	473** (372–819)
** Capacity-350 herds/day**	34 (7–106)	27 (4–141)	5,105 (646–25,614)	469 (372–757)
** Capacity-600 herds/day**	34 (7–103)	28 (4–133)	4,974 (656–27,233)	473 (372–746)
**lowPig**				
**Basic**	38 (7–113)	32 (4–158)	5,670 (588–25,611)	477 (372–743)
** Capacity-200 herds/day**	38 (8–115)	32 (4–156)	5,425*** (581–19,628)	479 (369–805)
** Capacity-350 herds/day**	37 (7–108)	31 (4–148)	5,488** (580–24,048)	477 (369–734)
** Capacity-600 herds/day**	37 (7–106)	30 (4–146)	5,702** (588–25,128)	474 (373–730)
**Sheep**				
** Basic**	30 (2–100)	20 (2–138)	3,341 (365–25,220)	449 (354–722)
** Capacity-200 herds/day**	29 (2–97)	20 (2–142)	3,045*** (343–16,811)	446 (354–775)
** Capacity-350 herds/day**	30 (2–104)	20 (2–132)	3,203 (365–24,355)	451 (352–720)
** Capacity-600 herds/day**	29 (2–101)	21 (2–136)	3,331 (365–26,558)	448 (354–714)

1 Epidemic duration is calculated from detection of the first herd in the epidemic to the last herd is depopulated.

2 Statistical significance level in comparison to the corresponding variable in the corresponding basic scenario (absence of a star represents a P-value ≥0.05, * represents a P-value <0.05, ** represents a P-value <0.01, and *** represents a P-value <0.001).

When the frequency of surveying herds that are located in overlapping surveillance zones was changed from once every 14 days to once every 7, 21 or 30 days, the number and proportion of herds located in overlapping surveillance zones were close to those observed in the corresponding basic scenario. Changing the frequency to 7 or 30 days, increased or decreased the number of surveillance visits, respectively, while there were no changes to the number of infected herds, the number of diagnosed herds from surveillance and epidemic duration and costs (Table 3).

**Table 3:** Median with (5^th^ and 95^th^ percentiles) of epidemic duration, number of infected herds, number of diagnosed herds from surveillance, number of surveillance visits and the total costs of the epidemic, using the basic scenario (**Basic**) that represent the EU and Danish control measures, when epidemics were initiated in cattle herds in high (**highCat**) and low (**lowCat**) cattle density area, swine herds in high (**highPig**) and low (**lowPig**) swine density area and in sheep herds (**sheep**); The influence of changing the frequency of surveying herds located in overlapping zones from every 14 days to every 7, 21 or 30 days is compared.

	Epidemic duration (days)^1^	Infected herds	Diagnosed herds from surveillance	Surveillance visits	Total costs (€×10^6^)
**highCat**					
** Basic**	45 (14–113)	56 (10–192)	7 (0–27)	11,122 (1,896–35,839)	522 (400–829)
** Survey every 7 days**	46 (14–117)	56 (9–197)	7 (1–23)	15,381***^2^ (1,967–40,248)	528 (397–822)
** Survey every 21 days**	47 (14–113)	59 (9–191)	7 (1–24)	10,298 (1,680–32,312)	534 (399–817)
** Survey every 30 days**	48 (14–110)	59 (9–185)	7 (1–24)	9,989*** (1,575–29,301)	536 (399–800)
**lowCat**					
** Basic**	57 (17–129)	77 (13–269)	10 (1–36)	12,746 (1,582–37,561)	558 (405–858)
** Survey every 7 days**	55 (17–126)	75 (13–259)	11 (1–37)	16,585*** (2,007–44,861)	548 (406–852)
** Survey every 21 days**	57 (17–129)	76 (14–259)	10 (1–38)	11,110** (1,489–33,343)	557 (403–836)
** Survey every 30 days**	61 (17–140)	79 (14–271)	10 (1–39)	10,634*** (1,420–32,248)	569 (403–882)
**highPig**					
** Basic**	33 (7–101)	27 (4–129)	3 (0–17)	4,852 (656–26,873)	468 (372–726)
** Survey every 7 days**	34 (7– 102)	28 (4–121)	3 (0–17)	6,571*** (755–31,191)	467 (372–719)
** Survey every 21 days**	34 (7–98)	28 (4–127)	3 (0–15)	4,549 (642–21,623)	469 (372–712)
** Survey every 30 days**	34 (7–97)	27 (4–127)	3 (0–15)	4,066** (642–20,197)	468 (372–710)
**lowPig**					
** Basic**	38 (7–113)	32 (4–158)	4 (0–22)	5,670 (588–25,611)	477 (372–743)
** Survey every 7 days**	37 (7–112)	30 (4–146)	4 (0–22)	7,454*** (658–33,284)	476 (370–753)
** Survey every 21 days**	41 (7–112)	33 (4–151)	4 (0–22)	5,276 (581–22,637)	487 (372–756)
** Survey every 30 days**	39 (7–113)	32 (4–142)	4 (0–18)	4,575** (581–21,385)	482 (372–748)
**Sheep**					
** Basic**	30 (2–100)	20 (2–138)	2 (0–19)	3,341 (365–25,220)	449 (354–722)
** Survey every 7 days**	28 (2–100)	19 (2–134)	2 (0–19)	3,969** (382–31,743)	445 (352–725)
** Survey every 21 days**	30 (2–108)	20 (2–140)	2 (0–17)	3,032*** (365–24,442)	449 (354–753)
** Survey every 30 days**	31 (2–105)	21 (2–140)	2 (0–18)	2,781* (365–21,207)	450 (354–734)

1 Epidemic duration is calculated from detection of the first herd in the epidemic to the last herd is depopulated.

2 Statistical significance level in comparison to the corresponding variable in the corresponding basic scenario (absence of a star represents a P-value ≥0.05, * represents a P-value <0.05, ** represents a P-value <0.01, and *** represents a P-value <0.001).

Increasing surveillance capacity from 450 to 600 herds per day and enlarging the protection and/or the surveillance zones would most often not affect epidemic duration, number of infected herds and total costs (Table 4), compared to the corresponding scenario using the default surveillance capacity (Table 1). However, more herds would be surveyed when surveillance capacity is increased (Table 4).

**Table 4:** Median with (5^th^ and 95^th^ percentiles) of epidemic duration, number of infected herds, number of surveillance visits and the total costs of the epidemic, following enlargements of the protection zone from 3 km to 5 km (**PZ5**) and surveillance zone from 10 km to 15 km (**SZ15**) or 20 km (**SZ20**), and a combination of these enlargements, and increasing surveillance capacity from 450 herds per day to 600 herds per day, when epidemics were initiated in cattle herds in high (**highCat**) and low (**lowCat**) cattle density area, swine herds in high (**highPig**) and low (**lowPig**) swine density area and in sheep herds (**sheep**).

	Epidemic duration (days)^1^	Infected herds	Surveillance visits	Total costs (€×10^6^)
**highCat**				
** PZ5**	44 (13–106)	55 (9–178)	12,287 (1,868–40,073)	525 (399–799)
** SZ15**	42 (13–99)	52 (8–167)	16,703*^2^ (2,842–46,513)	538 (399–852)
** SZ20**	40 (13–96)	48 (9–169)	19,941*** (3,569–51,503)	544 (407–944)
** PZ5+SZ15**	43 (13–97)	52 (10–162)	18,201** (3,164–45,972)	540 (404–823)
** PZ5+SZ20**	41 (13–89)	48 (9–144)	21,010*** (4,105–47,190)	557 (408–855)
**lowCat**				
** PZ5**	56 (18–136)	76 (13–264)	13,772 (1,904–45,936)	552 (405–868)
** SZ15**	51 (16–114)	68 (12–225)	17,192 (2,129–48,082)	557 (404–854)
** SZ20**	47* (15–106)	63 (12–215)	20,991* (2,747–53,332)	559* (413–947)
** PZ5+SZ15**	53 (17–117)	65 (12–242)	18,789* (2,786–50,217)	565 (412–860)
** PZ5+SZ20**	49 (16–115)	64 (12–212)	22,273*** (3,334–56,115)	568 (412–944)
**highPig**				
** PZ5**	35 (7–107)	28 (4–129)	5,715 (837–29,283)	473 (372–751)
** SZ15**	33 (7–92)	27 (4–108)	8,363 (1,276–34,616)	482 (376–734)
** SZ20**	32 (7–82)	25 (4–93)	10,647 (1,610–38,393)	485 (379–762)
** PZ5+SZ15**	33 (7–86)	26 (4–104)	8,467 (1,343–34,282)	480 (376–723)
** PZ5+SZ20**	32 (7–83)	25 (4–98)	11,121 (1,846–39,143)	491 (381–751)
**lowPig**				
** PZ5**	37 (7–110)	30 (4–158)	5,837 (737–30,835)	473 (273–559)
** SZ15**	33 (7–91)	28 (4–117)	8,351 (1,090–31,455)	474 (275–520)
** SZ20**	34 (7–89)	28 (4–111)	11,644* (1,610–39,374)	488 (278–561)
** PZ5+SZ15**	35 (8–102)	29 (4–129)	8,928 (1,230–36,426)	481 (275–566)
** PZ5+SZ20**	34 (8–90)	28 (4–118)	12,185** (1,772–38,772)	489 (282–552)
**Sheep**				
** PZ5**	30 (2–102)	20 (2–124)	3,740 (410–27,242)	449 (352–725)
** SZ15**	29 (2–88)	19 (2–116)	5,409 (638–35,592)	456 (360–715)
** SZ20**	28 (3–86)	18 (2–104)	7,896 (795–40,161)	462 (361–763)
** PZ5+SZ15**	29 (2–88)	18 (2–123)	5,651 (702–36,926)	454 (358–727)
** PZ5+SZ20**	28 (3–90)	18 (2–116)	7,954 (837–43,499)	461 (363–792)

1 Epidemic duration is calculated from detection of the first herd in the epidemic to the last herd is depopulated.

2 Statistical significance level in comparison to the corresponding variable and scenario in Table 1 (absence of a star represents a P-value ≥0.05, * represents a P-value <0.05, ** represents a P-value <0.01, and *** represents a P-value <0.001).

## Discussion

Prior to the UK 2001 epidemic, the contingency plan of the Ministry of Agriculture, Forestry and Fisheries for notifiable diseases included that in case of a severe case scenario of spread of a specific exotic disease, the UK would need 235 veterinary officers. In a more extensive outbreak, the number of staff needed might rise to 300 [22]. Nonetheless, during the outbreak, 2,500 temporary veterinary inspectors were assigned, with nearly 70 from abroad, and a further 700 foreign government veterinarians and personnel assisted on temporary basis [22]. This reflects the importance of assessing whether available resources are sufficient to control an epidemic of FMD, in order to improve the contingency plan. The current study shows that the available resources for clinical surveillance, in case of an FMD outbreak in Denmark, seem to be sufficient to survey herds within the protection and surveillance zones on time.

Regardless the type of index herd that was used to initiate the epidemics, reducing surveillance capacity did not change the epidemic duration and the number of infected herds. Nonetheless, it resulted in a larger costs and variability around the predicted costs in most situations, compared to the corresponding basic scenario. It also resulted in a fewer number of surveillance visits (Table 2). When surveillance capacity was reduced to 200 herds per day, a herd would have to wait few days before it could be surveyed (Figure 3). This delay would apparently not result in further spread of the disease, as the number of infected herds was not different from the corresponding basic scenario. Nonetheless, lower capacity might result in more variability in disease spread, and thus large epidemics might occur more frequently, as shown from the 95% percentiles (Table 2). At least, 8 days should elapse between two surveillance visits (see materials and methods). When resources were reduced to 200 herds per day, long delay time occurred (Figure 3), and therefore a herd could be set in the queue for a new surveillance visit, while the previous visit was not yet executed. In such cases, the model was set to execute only the first visit, which resulted in fewer number of surveillance visits (Table 2).

On the other hand, increasing surveillance capacity does not seem to affect the epidemic course (Table 2). This indicates that the estimated surveillance capacity in Denmark, under the modelled regulations, is sufficient to fulfill the EU and Danish regulations of surveying herds that are within the protection and surveillance zones without delays. However, when the surveillance zone was enlarged, the surveillance capacity was normally not sufficient to survey herds on time, when large epidemics occurred (Figure 2). Thus extra resources might be needed when the Veterinary Authorities consider enlarging the surveillance zone.

When the surveillance capacity was increased in scenarios with enlarged zones, more herds were surveyed, as a result of shorter waiting times for surveillance visits. Repeated visits that were not executed due to the lack of resources in the basic scenario were now executed, due to the availability of more resources. Nevertheless, enlargement of the zones combined with extra surveillance capacity, in most situations, did not minimize the economic losses of the simulated epidemics (Table 4).

Overlapping zones are expected to occur during an outbreak. It is important to determine, how often herds should be re-surveyed, in order to optimize FMD control, when new zones are created, including herds already in other zones. As explained earlier, we assumed that herds within overlapping surveillance zones would be surveyed every 14 days, as long as they are in overlapping zones. This assumption was based on expert knowledge from the Veterinary Authorities and their experience with other disease outbreaks. Nonetheless, a sensitivity analysis was conducted by changing this value to 7, 21 and 30 days. Increasing the surveillance frequency to every 7 days would not change the course of the epidemic, nor the number of detected herds through surveillance (Table 3). Furthermore, reducing it to every 21 or 30 days would not change the number of herds detected through surveillance nor the total costs (Table 3). Generally, this indicates that the first surveillance visit seems to be important to detect herds early through surveillance. Repeated visits for these herds do not seem to be necessary, and thus can be minimized. It is important to mention though, that the model assumes that the surveillance teams are highly effective in finding clinical signs if present. During an outbreak, veterinarians will be very aware of the possibility of infection, and thus they will most likely find existing clinical signs. Given the availability of resources, the Veterinary Authorities would most likely re-survey herds as frequently as possible, in order to convince the World Organization of Animal Health (OIE) and EU member states of the sufficiency of the applied measures, to regain the free status as fast as possible.

Enlarging the protection zone was as good as the corresponding basic scenario, in terms of epidemic duration, number of affected herds and total epidemic costs. Although it resulted in larger number of surveillance visits compared to the corresponding basic scenario, the resources were usually sufficient to survey herds on time. Furthermore, enlarging the protection zone was a cheaper strategy than the corresponding basic scenario, in case of large epidemics as indicated by the 95^th^ percentile of the total costs of this scenario, regardless the index herd type that was used to initiate the epidemics (Table 1). Enlarging the protection zone was as good as the basic scenario in median size epidemics, but it included the advantage of minimizing economic losses in case of large epidemics, which makes it a better strategy than the basic scenario.

In certain situations, enlarging the surveillance zones resulted in shorter epidemic duration and lower number of infected herds (Table 1). However, it resulted frequently in larger number of surveillance visits (Table 1), and hence to extra delays, before herds can be visited (Figure 2). It also resulted in larger total costs compared to the corresponding basic scenario. This was due to the larger number of surveillance visits, which lead to higher direct costs (Table 1). Important to mention that it was assumed in the economic calculations that only herds outside the surveillance zones can export products to EU countries, without price reduction [10]. This means that enlargement of the surveillance zones would result in larger economic damage due to larger export loss to the EU countries (Table 1). Moreover, it was more often necessary to prolong the duration of the zones, when the surveillance zones were enlarged compared to the basic scenario. Longer zone duration means larger economic damage due to larger export loss. Generally, this means that the potential gain from shorter epidemic duration and fewer infected herds, caused by the enlarged surveillance zone, would not pay off the economic damage due to the higher costs. Shorter epidemic duration and fewer infected herds might actually include an advantage of reducing the risk of losing markets. In case of an epidemic, countries that import livestock and/or livestock products from Denmark might either find other suppliers and completely stop imports from Denmark, or might continue imports, following the end of the restriction on export, but with a lesser extent than before the epidemic. Although it is difficult to predict the reaction of foreign markets in case of an epidemic [10], [23], the risk of losing markets would probably positively correlate with epidemic duration. Thus the economic outcomes might differ depending on the reaction of the importing countries.

The results shown in our study are influenced by the herd structure in Denmark and the large export of especially pigs and pig products. Therefore, the effect of enlarged zone sizes might be different in other countries. Furthermore, in this study we focused on zone size and surveillance capacity with the basic control strategy. In future work, it will be interesting to investigate the effect of changes on, for example, pre-emptive depopulation or emergency vaccination.

## Conclusions

The available resources for clinical surveillance, in case of an FMD outbreak in Denmark, are sufficient to survey herds in the protection and surveillance zones within the first week of the zones' establishment, under EU and Danish control regulations. However, when enlarging the surveillance zone is considered, extra resources may be needed, in order to survey herds on time. Generally, enlargement of the protection zone seems to be a better option than the basic scenario. Enlarging the surveillance zone may reduce epidemic duration and the number of affected herds. However, reduction of the economic losses would not be expected. Extra resources for clinical surveillance do not minimize the total costs of the epidemic when the protection and/or surveillance zones are enlarged. Fewer resources may result in larger and costlier epidemics.
